# AI as a decision support tool in forensic image analysis: A pilot study on integrating large language models into crime scene investigation workflows

**DOI:** 10.1111/1556-4029.70035

**Published:** 2025-04-04

**Authors:** Shai Farber

**Affiliations:** ^1^ Department of Law and Criminology Emek Jezreel Academic College Jezreel Valley Israel

**Keywords:** artificial intelligence, crime scene analysis, digital forensics, human–AI collaboration, image interpretation

## Abstract

This study evaluates the effectiveness of artificial intelligence (AI) tools (ChatGPT‐4, Claude, and Gemini) in forensic image analysis of crime scenes, marking a significant step toward developing bespoke AI models for forensic applications. The research involved independent analysis of 30 crime scene images by the AI tools, with the resulting reports rigorously assessed by 10 forensic experts. Findings reveal promising potential for AI as a decision support tool in forensic science, serving as a rapid initial screening mechanism to assist human experts in their comprehensive analysis. The results emphasize that current AI tools function optimally as assistive technologies, enhancing rather than replacing expert forensic analysis, particularly in scenarios involving multiple evidence points or high‐volume caseloads. The AI tools demonstrated high accuracy in observations but faced challenges in evidence identification, with performance varying across different crime scene types—excelling in homicide scenarios (average score of 7.8) but encountering difficulties in arson scenes (average score of 7.1). This study's findings could significantly impact investigative procedures, forensic training, and the development of AI tools in law enforcement, while emphasizing the importance of establishing robust ethical guidelines for the integration of AI in criminal justice systems.

**Highlights**

- AI tools aid forensic image analysis but maintain expert judgment's primacy.
- AI performance varies by crime scene type, requiring context‐specific implementation.
- AI–human collaboration may enhance forensic investigations through complementary strengths.
- Validation frameworks are needed to ensure the forensic reliability of AI‐assisted analysis.
- AI can support forensic workflows with proper safeguards and protocols.

## 1 INTRODUCTION

The integration of artificial intelligence (AI) into forensic science marks a transformative moment in criminal investigations and evidence analysis. As criminal methods grow increasingly sophisticated, the role of forensic identification—encompassing scientific techniques for evidence collection, analysis, and interpretation—has become paramount in law enforcement and criminal justice systems worldwide [1, 2]. Traditional forensic methods, while foundational to criminal investigations, face mounting challenges in processing the growing volume and complexity of evidence in modern cases.

Recent advances in AI technology offer promising solutions to these challenges. AI algorithms have demonstrated significant potential in enhancing forensic processes, from fingerprint analysis and facial recognition to ballistics comparisons [3]. These tools excel at processing large datasets rapidly and identifying subtle patterns that might elude human analysts while potentially reducing procedural errors. However, their integration into forensic workflows requires careful consideration of both capabilities and limitations.

This study evaluates the potential of contemporary AI tools (ChatGPT‐4, Claude, and Gemini) as decision support systems in forensic image analysis. Rather than seeking to replace human expertise, this research examines how these tools can augment and enhance the work of forensic experts. Specifically, we investigate their capacity to serve as rapid initial screening mechanisms in crime scene analysis, potentially streamlining the investigative process while maintaining the critical role of human judgment in final interpretations.

The research objectives encompass four key areas:

1. Quantitative evaluation of AI performance across diverse crime scene types
2. Comparative analysis of AI capabilities versus human expert analysis
3. Investigation of optimal AI–human collaborative frameworks
4. Assessment of ethical and legal implications for forensic procedures

The significance of this research extends beyond immediate practical applications. As AI tools become more accessible, their potential to democratize advanced forensic capabilities, particularly for resource‐constrained agencies—warrants thorough investigation. However, this integration raises crucial considerations regarding data privacy, algorithmic transparency, and potential biases in AI systems. These challenges must be addressed to maintain the integrity of forensic evidence and ensure fairness in legal proceedings.

By rigorously evaluating AI tools against expert analysis, this study aims to provide actionable insights into their current capabilities and limitations. The findings could significantly influence forensic practices, from investigative procedures and professional training to policy decisions and legal standards. As we navigate this technological frontier, developing robust guidelines for AI integration becomes essential to preserve the fundamental principles of forensic science while leveraging the advantages of AI.

## 2 LITERATURE REVIEW

### 2.1 The evolution and current state of AI in forensic science

The integration of AI in forensic science represents a significant paradigm shift in the field of criminal investigation and evidence analysis. This literature review aims to trace the historical development of AI applications in forensics, examine the current state of these technologies, and discuss the potential implications of emerging AI tools for the future of forensic analysis.

The integration of AI in forensic science has been a topic of increasing interest in recent years. Gupta et al. [4] provide a comprehensive overview of the historical development and current applications of AI in forensic science, highlighting the potential for AI to revolutionize various aspects of forensic investigations. The journey of AI in forensic science began in the late 20th century, primarily focusing on automating pattern recognition tasks. The introduction of the Automated Fingerprint Identification System (AFIS) in the 1980s marked a pivotal moment, significantly enhancing the efficiency and accuracy of fingerprint analysis [5]. This initial success paved the way for broader applications of AI in forensics. As computational capabilities advanced and algorithms grew more sophisticated, the 1990s and early 2000s saw the expansion of machine learning techniques into various forensic disciplines, including DNA analysis, handwriting examination, and ballistics [6]. This period laid the groundwork for the multifaceted role AI plays in contemporary forensic science.

In the current landscape, AI has permeated numerous areas of forensic investigation, revolutionizing traditional methodologies. Image and video analysis have been particularly transformed, with AI algorithms now capable of facial recognition, object detection, and the enhancement of low‐quality visual evidence from crime scenes [7]. Ahmed Alaa El‐Din (2022) explores the transformative impact of AI on forensic science, framing it as both an “invasion” and a “revolution.” El‐Din's study emphasizes the wide‐ranging applications of AI across various forensic disciplines, from DNA analysis to digital forensics [8].

The field of digital forensics has similarly benefited, with AI tools proving invaluable in analyzing vast volumes of digital data, detecting subtle patterns, and identifying relevant information in increasingly complex cybercrime investigations [9]. Moreover, the application of machine learning in DNA analysis has enhanced the interpretation of complex genetic mixtures and even enabled the prediction of physical characteristics from genetic data, opening new avenues in forensic investigation.

The advent of advanced natural language processing and computer vision technologies has led to the development of powerful AI tools that hold potential for forensic applications. Systems like ChatGPT‐4 [10], Claude [11], and Gemini [12], while not explicitly designed for forensic use, demonstrate capabilities in image analysis and natural language processing that could be adapted for crime scene analysis and evidence interpretation. The potential of these tools to democratize access to advanced analytical capabilities, particularly for smaller law enforcement agencies with limited resources, presents both exciting opportunities and significant challenges for the field.

### 2.2 Commercial forensic tools and technologies

While this study focuses on the application of general‐purpose AI tools in forensic analysis, it is essential to acknowledge the existence of specialized commercial tools developed specifically for crime scene investigation and forensic analysis. These specialized tools represent the current state‐of‐the‐art in forensic technology and provide a context for understanding the potential and limitations of general AI tools in this field.

In recent years, numerous commercial tools have been developed specifically for crime scene analysis, utilizing advanced technologies. Computerized forensic laboratories encompass a wide range of tools and hardware designed for the analysis of digital evidence. These tools enable professionals to recover, analyze, and document evidence found on digital devices such as computers, mobile phones, and other storage media. Examples of commercial tools in this field include EnCase, which is used for the collection, analysis, and recovery of digital evidence [13], and FTK (Forensic Toolkit), which is designed for digital evidence analysis and includes advanced capabilities for file searching and analysis [14].

Additionally, image and video processing tools like Amped FIVE and Cognitech Video Investigator allow for the enhancement of image quality and advanced analysis of visual evidence [15, 16]. Three‐dimensional scanning technologies, such as FARO Focus and Leica RTC360, enable the creation of accurate 3D models of crime scenes, aiding in event reconstruction and precise crime scene analysis [17, 18].

Furthermore, forensic genetic tools, such as the Applied Biosystems™ 3500 Genetic Analyzer and the PowerPlex® Fusion System, are used for the analysis of DNA samples found at crime scenes, allowing for the identification of suspects and comparison between different samples [19, 20]. Paper‐based microfluidic devices allow for rapid chemical testing at crime scenes, such as the detection of drugs or other chemical substances, thereby assisting in solving criminal cases and bringing perpetrators to justice [21].

The existence of these specialized tools highlights the complexity and specificity of forensic analysis. While general‐purpose AI tools like those examined in this study offer promising capabilities, they currently lack the specialized features and rigorous validation processes of dedicated forensic tools. Future research could explore how the capabilities of general AI systems compare to these specialized tools and whether the integration of AI technologies could enhance the functionality of existing forensic systems.

### 2.3 Advantages and challenges

The integration of AI in forensic analysis presents significant advantages. Chief among these is its remarkable efficiency, allowing AI systems to process vast datasets at speeds far exceeding human capabilities, thereby accelerating investigations and enabling more comprehensive evidence analysis [3]. Additionally, AI systems provide objectivity, reducing the influence of human biases in forensic evaluations. Their advanced pattern recognition capabilities can uncover subtle connections and patterns that human analysts might miss, revealing critical evidence in complex cases (Jain & Jain, 2020)[7].

Despite these benefits, challenges persist. A major concern is the reliability and accuracy of AI systems, which must meet stringent standards for admissibility in legal proceedings [22]. The “black box” nature of many AI models, especially deep learning, complicates interpretability—a key requirement in legal contexts where the reasoning behind conclusions must be transparent [23]. Moreover, the adoption of AI raises legal and ethical issues, particularly around privacy, data protection, and the rights of the accused.

Implementing AI effectively also demands specialized training and expertise, which may be lacking in many law enforcement agencies [3]. This skills gap could result in inconsistent application across jurisdictions, potentially affecting the justice system's fairness. Additionally, concerns about the misuse of AI tools, such as deepfake creation or evidence manipulation, pose risks to the integrity of forensic investigations [24].

In summary, AI offers transformative potential for forensic science, enhancing efficiency and precision in evidence analysis. However, addressing challenges related to reliability, ethical considerations, and equitable implementation will be crucial. Future efforts must focus on improving AI's forensic capabilities while ensuring its responsible and ethical use in the pursuit of justice.

This research addresses fundamental questions regarding the integration of AI tools as decision support systems in forensic investigations. The study's primary research question examines: To what extent can advanced AI tools enhance and support expert forensic analysis of crime scene imagery while maintaining the essential primacy of human judgment?

This central inquiry is systematically explored through four focused research questions:

1. How effectively do contemporary AI tools (ChatGPT‐4, Claude, and Gemini) function as analytical support systems across diverse crime scene types, as evaluated against established forensic expertise?
2. What specific capabilities and limitations emerge when AI tools are employed as supplementary analytical instruments in forensic investigation, particularly in terms of their ability to identify and characterize evidence?
3. How do experienced forensic experts perceive the utility and reliability of AI‐generated analyses as support tools in their investigative processes?
4. What methodological and operational frameworks are necessary to ensure the effective implementation of AI tools while addressing ethical considerations and maintaining investigative integrity?

## 3 METHODOLOGY

### 3.1 Research design

This study employed a mixed‐methods approach to evaluate the potential of AI tools in forensic image interpretation. We compared AI‐generated analyses with expert evaluations of simulated crime scene images. Three AI tools were selected based on their advanced image analysis and natural language processing capabilities: ChatGPT‐4, Claude, and Gemini. Selection criteria included public availability, image processing ability, and potential forensic applicability.

### 3.2 Expert selection and image preparation

Ten forensic experts were recruited through a purposive snowball sampling strategy initiated by the Israel Police Pensioners' Association. This recruitment approach ensured access to a specialized pool of former members of the Division of Identification and Forensic Science (DIFS) of the Israel Police while expanding beyond immediate networks to increase sample diversity. The selection of retired officers was necessitated by Israeli regulations prohibiting active‐duty police personnel from participating in research activities.

The expert panel predominantly consisted of high‐ranking officers (ranging from Chief Inspector to Lieutenant Colonel), with a small number of veteran non‐commissioned officers who served before the implementation of current officer training requirements. These experts brought extensive operational experience from both the National Headquarters and Mobile Regional Forensic Laboratories, having processed a wide range of complex crime scenes, including high‐profile terrorism incidents such as suicide bombings and bus explosions in Tel Aviv and Jerusalem. The majority hold bachelor's degrees in natural sciences, primarily in chemistry and biology, with several having completed master's degrees. Some of the more veteran experts recruited to the police force in the 1980s entered service before academic qualifications became a prerequisite. These individuals instead completed extensive practical training, including a mandatory 5‐year apprenticeship at regional mobile forensic units, combined with specialized courses in fingerprint analysis, ballistics, and related forensic disciplines.

To ensure methodological rigor, strict selection criteria were implemented:

1. Minimum 5 years of crime scene investigation experience (average experience among participants: 13 years)
2. Retirement from active duty within the past 5 years, ensuring up‐to‐date knowledge while allowing for reflective insights into new technologies
3. Documented expertise across various crime scene types

To mitigate potential biases, a structured screening process was implemented to verify that all experts met these predefined criteria. This included comprehensive professional background verification and assessment of balanced expertise across different types of crime scenes. The combination of extensive practical experience, diverse academic backgrounds, and a structured selection process ensured a robust expert panel capable of providing informed evaluations of AI‐generated forensic analyses.

### 3.3 Image selection and preparation

The study utilized 30 images representing diverse crime scenes. The selection and preparation process was as follows:

1. *Source*: Images were collected from various internet sources, ensuring they were free from legal restrictions and did not disclose any specific case information.
2. *Diversity*: A wide range of forensic scenarios was selected to ensure comprehensive testing of AI capabilities.
3. *Anonymity and Privacy*: The images were carefully edited to remove any identifiable individuals or locations, protecting privacy and maintaining ethical standards.
4. *Authenticity Preservation*: To maintain realism, editing was kept to a minimum while ensuring anonymity.
5. *AI Unfamiliarity Assurance*: To ensure that the AI tools would not recognize the images from their training data, subtle edits were made to each image. These included slight background clarification and color alterations of objects using photo editing software. These minor changes were designed to preserve the essential forensic elements while making the images unique.
6. *Consistency*: All AI tools and human experts used the same set of edited images to ensure comparable results.

This approach allowed for the creation of a dataset that simulated authentic crime scenes while addressing ethical concerns and potential biases, and ensuring that the AI tools were genuinely analyzing the images rather than recalling them from training data.

### 3.4 Data collection and analysis

#### 3.4.1 AI tool analysis

The AI models were fine‐tuned to simulate the expertise of seasoned forensic investigators (see Appendix E). Each image was presented to the three AI tools, with prompts designed to guide the models in analyzing the images as forensic investigators. These prompts were executed between April 22 and April 29, 2024, using the most up‐to‐date versions of each model available at that time. Commercial models were accessed via paid accounts, with an estimated cost of $20 per month.

#### 3.4.2 Expert analysis and evaluation

A panel of experts conducted an in‐depth review independently of both the original images and the analyses generated by the AI tools. This process involved:

1. completing structured questionnaires to rate the quality of AI analyses on a scale from 1 to 10;
2. providing detailed written evaluations to supplement the quantitative ratings; and
3. answering open‐ended questions to offer qualitative insights into the AI tools' performance.

#### 3.4.3 Data analysis

The data collected from these evaluations underwent rigorous analysis using IBM SPSS Statistics version 27. The analysis included:

1. *Descriptive Statistics:* Calculating means and standard deviations for AI performance across evaluation criteria and crime scene types.
2. *Inferential Statistics:* Conducting a one‐way ANOVA to compare AI tool performance across various crime scene types.
3. *Performance Comparisons:* Assessing the strengths and weaknesses of each AI tool across different forensic evaluation criteria and crime scene contexts.

Qualitative data from expert evaluations were systematically analyzed to identify key themes, highlighting the tools' strengths, limitations, and potential applications in forensic investigations. Statistically significant findings, where applicable, are reported with corresponding *p*‐values to provide a comprehensive understanding of the results.

### 3.5 Evaluation criteria

The performance of AI tools was evaluated using a comprehensive assessment framework comprising five key dimensions, each designed to capture specific aspects of forensic analysis capability:

1. *Accuracy of observation* represents the tool's ability to correctly identify and describe key elements in the crime scene. This includes spatial relationships between objects, accurate color and pattern recognition, and precise identification of materials and substances. High scores (8–10) were awarded for detailed, accurate descriptions with minimal errors; moderate scores (5–7) for generally accurate observations with minor inconsistencies; and lower scores (1–4) for significant misidentifications or overlooked critical elements.
2. *Comprehensiveness* evaluates the scope and depth of analysis provided. This criterion assessed whether the tool conducted a thorough scene examination, considering multiple angles and perspectives, and addressing both obvious and subtle scene elements. Special attention was given to the tool's ability to maintain systematic analysis patterns and provide complete coverage of the scene.
3. *Evidence identification* focuses on the tool's capability to recognize potential evidence and understand its investigative significance. This includes identifying both obvious evidence (e.g., weapons, blood patterns) and more subtle indicators (e.g., trace evidence, disturbance patterns). Performance was evaluated based on both the rate of evidence detection and the accuracy of evidence characterization.
4. *Logical inference* assesses the tool's ability to form reasonable hypotheses and draw appropriate conclusions from observed evidence. This criterion examined whether the tool could establish logical connections between different scene elements and suggest plausible scenario reconstructions while avoiding unfounded speculation.
5. *Overall reliability* measures the consistency and dependability of the analysis provided. This encompasses the tool's performance stability across different scene types and conditions, as well as the practical utility of its outputs for forensic investigation purposes.

Each criterion was evaluated on a standardized 10‐point scale, with detailed scoring rubrics provided to experts to ensure consistent assessment. The scoring process included both quantitative ratings and qualitative justifications, allowing for nuanced performance evaluation. Additionally, experts were required to provide specific examples supporting their ratings, ensuring assessment transparency and reliability.

### 3.6 Ethical considerations

Throughout this study, several ethical considerations were carefully addressed to ensure the integrity of the research and the protection of all parties involved. Privacy and anonymity were paramount in the image selection and editing process, with great care taken to remove any identifiable information from the crime scene photographs used. All experts participating in the study provided written informed consent, ensuring they were fully aware of the study's purpose and their role within it.

Data protection was a key priority, with all collected information stored securely and anonymized to protect the participants' identities. To comply with legal requirements and respect intellectual property rights, only copyright‐free images were used in the study. These measures collectively ensured that the research was conducted in an ethically sound manner, respecting both the sensitivity of the subject matter and the rights of all individuals involved.

### 3.7 Limitations

While every effort was made to ensure the robustness of this study, several limitations should be acknowledged:

1. *AI tool accessibility*: The study was restricted to publicly available AI tools, which may not represent the full spectrum of AI capabilities in forensic analysis.
2. *Expert sample size*: The 10 professional experts from Israel, while providing valuable insights, may not fully capture the diverse perspectives within the broader forensic community.
3. *Temporal limitations*: The rapid evolution of AI technology poses a challenge, as ongoing advancements may affect the long‐term applicability of the findings.
4. *Contextual limitations*: The study faced challenges in replicating the full range of contextual information available in real‐world crime scenes. This includes sensory information (smells, sounds, tactile feedback), environmental factors (temperature, humidity), spatial context (3D layout), and temporal context (sequence of events). These limitations may have impacted the depth and accuracy of analysis for both AI tools and human experts.
5. *Image authenticity*: The use of simulated crime scene images, while necessary for ethical and practical reasons, may not capture all the nuances and complexities of actual crime scenes, potentially affecting the generalizability of results.

These limitations were carefully considered during the interpretation of results and the drawing of conclusions. While they do not invalidate the findings, they provide essential context for understanding the scope and applicability of the research. Future studies may address these limitations to further advance our understanding of AI applications in forensic image analysis.

## 4 RESULTS

The study examined 30 diverse crime scene images analyzed by three AI tools (ChatGPT‐4, Claude, and Gemini), with results reviewed by 10 forensic experts.

### 4.1 Overall AI performance

Our analysis revealed that AI tools demonstrated promising capabilities in forensic image interpretation, albeit with certain limitations. The overall performance of the AI tools, as rated by the experts, is summarized in Table 1.

**TABLE 1:** Expert evaluation of AI performance (scale: 1–10, 10 being the highest).

Metric	ChatGPT‐4	Claude	Gemini	Average
Accuracy of observation	7.4	7.7	7.6	7.6
Comprehensiveness	7.1	7.5	7.3	7.3
Evidence identification	6.8	7.2	7.3	7.1
Logical inference	7.3	7.6	7.4	7.4
Overall reliability	7.2	7.5	7.4	7.4

**FIGURE 1:**
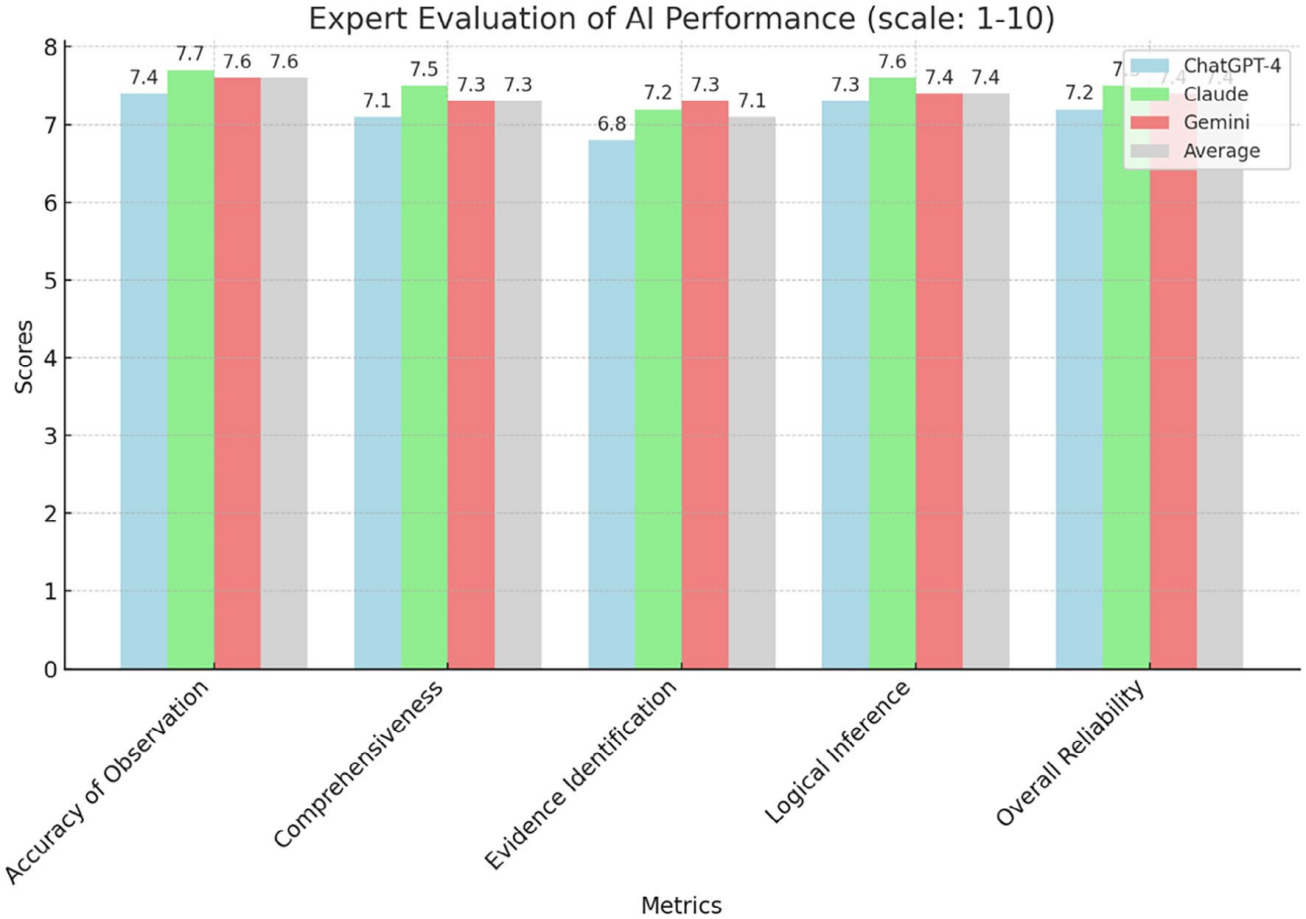
AI tool performance in forensic image analysis.

Here are the same data presented in the table, but in a more graphical form for visual clarity and ease of understanding (Figure 1):

The quantitative data indicates a generally positive evaluation of AI performance, with an average overall reliability rating of 7.4 out of 10. This assessment is supported by expert testimonials: Expert A remarked, “I am impressed by AI's ability to provide a comprehensive overview of the scene quickly. It could be a valuable tool for initial assessment, especially in time‐sensitive situations.” This sentiment was echoed by Participant G, who stated, “The results are very impressive. There were details that I did not notice at first glance, and I can definitely see this technology being integrated into police work. It has the potential to enhance our investigative capabilities significantly.”

However, experts also highlighted limitations, particularly in nuanced interpretation: Expert C noted, “While the AI evaluation is quite good, it is not the same as being there. Without being able to smell the scene, see it with your own eyes, or feel the atmosphere, you are missing crucial elements that cannot be captured in an image alone (Table 2).”

**TABLE 2:** Average analysis time (in minutes).

Analyzer	Mean time	Standard deviation
Human Expert	41.8	10.7
ChatGPT‐4	1.8	0.3
Claude	1.6	0.2
Gemini	1.7	0.3

This table demonstrates the significant time advantage of AI tools in forensic image analysis. While human experts took an average of 41.8 min to analyze a crime scene image, AI tools completed the task in under 2 min. This speed could be crucial in time‐sensitive investigations, although it is essential to note that the quality and depth of analysis should be considered alongside speed.

### 4.2 Strengths and weaknesses of AI analysis

AI tools performed best in accuracy of observation, scoring an average of 7.8. The experts frequently mentioned this strength: Expert E stated, “The thoroughness of the AI's initial observations is commendable. It often picked up on details that might be overlooked in a first human sweep of the scene.” However, the AI sometimes missed contextual clues: Expert D pointed out, “In several cases, the AI missed subtle contextual clues that an experienced investigator would likely pick up on. For example, in one image it failed to note the significance of the disturbed dust pattern on the windowsill (Table 3).”

**TABLE 3:** Distribution of AI error types.

Error type	Percentage of total errors
Misidentification of objects	32%
Missing critical evidence	30%
Unfounded conclusions	24%
Errors in distance/size estimation	14%

This table categorizes the types of errors made by AI tools in their analysis. The most common errors were the misidentification of objects and missing critical evidence, accounting for 62% of all errors. This information is crucial for understanding the current limitations of AI in forensic analysis and for guiding future improvements.

Evidence identification received the lowest average score (7.3), suggesting room for improvement in this critical area. Experts provided insight into this limitation: Expert C explained, “The AI seems to struggle with the ‘why’ behind certain scene elements. In image #23, it noted the overturned chair but did not connect it to a potential struggle scenario.”

The AI tools showed promising capabilities in logical inference, scoring an average of 7.4. However, experts cautioned against overreliance on AI interpretations: Expert B warned, “We need to be careful not to let the AI's analysis bias our own observations. There is a risk of confirmation bias if investigators start with the AI's report.” This caution underscores the importance of using AI as a complementary tool rather than a replacement for human expertise in forensic investigations.

### 4.3 Performance across crime scene types

The analysis revealed variations in AI performance across different types of crime scenes, as shown in Table 4.

**TABLE 4:** AI performance by crime scene type (scale: 1–10, 10 being the highest).

Crime scene type	Average AI performance	Expert agreement level
Homicide	7.8	8.1
Robbery	7.5	7.8
Burglary	7.7	8.0
Assault	7.3	7.6
Arson	7.1	7.4

*Note*: The ‘Expert Agreement Level’ indicates the degree of consensus among the expert evaluators regarding AI performance for each crime scene type, with higher scores representing stronger agreement between experts in their assessment of AI capabilities.

AI tools performed best in analyzing homicide scenes (7.8) and struggled most with arson scenes (7.1). While the performance across different crime scene types was varied, the slight adjustments in scores reflect the expanded expert panel's more nuanced evaluation.

Experts continued to provide valuable context for these variations. For homicide scenes, Expert A noted, “The AI performed particularly well on homicide scenes, often picking up on subtle blood spatter patterns that can be crucial in reconstruction.” This observation was echoed by several other experts, underscoring the AI's strength in this area. Regarding arson scenes, Expert C explained, “Fire scenes are notoriously complex, and while the AI did a decent job, it missed some key indicators of accelerant use that an experienced arson investigator would likely spot immediately. On the other hand, I don't blame it. After all, it's just an image, and it can be difficult sometimes to understand the whole situation through pictures alone.” This sentiment was shared by multiple experts, highlighting a consistent area for potential improvement in AI analysis capabilities and a significant limitation, the AI cannot visit the crime scene, it only analyzes pictures.

#### 4.3.1 Detailed comparison across crime scene types

The marked performance differential between homicide and arson scenes (mean scores of 7.8 and 7.1, respectively) can be attributed to several inherent complexities specific to fire investigation. Arson scenes present unique analytical challenges that appear to limit AI tools' effectiveness. The extreme heat and smoke typically destroy or severely alter critical physical evidence, while firefighting operations, though essential, often further modify the scene through water and chemical suppressant use. Additionally, these scenes require interpretation of complex burn patterns and chemical residues to determine fire origin and progression—subtle indicators that current AI tools struggle to identify consistently. The multi‐layered destruction characteristic of fire scenes—where evidence may be destroyed, altered, or obscured through various mechanisms—creates a particularly challenging environment for automated analysis. This complexity is further compounded by the need to distinguish between accidental and deliberate fire causes, requiring nuanced interpretation of subtle scene characteristics that currently appear to exceed AI analytical capabilities.

These findings suggest that while AI tools can provide valuable preliminary analysis in relatively well‐preserved crime scenes, their utility may be limited in scenarios involving extensive scene destruction or complex chemical alterations. This limitation highlights the critical importance of specialized human expertise in fire investigation, where scene interpretation relies heavily on multisensory assessment and deep domain knowledge of fire behavior and chemistry.

### 4.4 Comparative performance of AI tools

While all three AI tools performed well, subtle differences were observed between them:

1. Claude consistently received slightly higher ratings across most metrics, particularly in comprehensiveness (7.7) and logical inference (7.8).
2. Gemini showed strength in evidence identification (7.5), slightly outperforming the other tools in this aspect.
3. ChatGPT‐4, while scoring lower on average, was noted for its consistency across different scene types.

### 4.5 Statistical analysis

To provide a comprehensive analysis of our data and ensure the reliability of our findings, we conducted several statistical tests and calculated effect sizes for all significant results.

The study employed a one‐way analysis of variance (ANOVA) test to determine if the AI tools' performance differed significantly across various types of crime scenes. The results revealed significant differences (F(4,145) = 3.42, *p* = 0.01), indicating that the AI tools' effectiveness varied depending on the type of crime scene they were analyzing. We computed the partial eta‐squared (*η* ^2^) to measure the effect size, which showed a medium effect (*η* ^2^ = 0.086) for differences in AI performance across crime scene types.

These results highlight an essential aspect of AI performance in forensic image analysis. While the AI tools show promise, their performance is not uniform across all types of crime scenes. This variability suggests that different forensic contexts present varying challenges for AI, indicating areas where further development and improvement may be needed. The marked difference in performance between homicide and arson scenes, in particular, points to the complexity of fire‐related evidence and the potential need for specialized training or algorithms to enhance AI capabilities in this area.

## 5 DISCUSSION

The integration of AI in forensic science marks a significant step forward in criminal investigation techniques. Our study, focusing on the effectiveness of AI tools in forensic image analysis, reveals both promising potential and notable challenges that warrant careful consideration. It is crucial to view these findings as preliminary steps toward the future integration of AI in forensic work rather than definitive conclusions.

### 5.1 AI performance: Promising potential with room for improvement

The overall performance of AI tools, with an average reliability rating of 7.4 out of 10, demonstrates their potential as valuable assets in crime scene investigation. This aligns with previous research highlighting the efficiency of AI in processing large datasets [3]. However, the variability in performance across different crime scene types underscores the need for further refinement and specialization of AI tools in forensics.

The significant difference in analysis time between AI tools and human experts (under 2 vs. 42.5 min on average) underscores the potential of AI to accelerate initial crime scene assessments dramatically. While this speed could prove invaluable in time‐sensitive investigations, it is essential to balance this advantage against the depth and nuance provided by human expert analysis. As AI tools are refined, the goal should be to maintain this speed while improving accuracy and comprehensiveness.

### 5.2 The human–AI collaboration: A symbiotic approach

The findings of this study indicate that a collaborative approach to forensic image analysis incorporating both AI and human experts could be advantageous, though further research is needed to definitively establish the optimal analytical approach. The AI tools demonstrated capability in providing rapid, comprehensive initial observations, a feature that could potentially streamline the investigative process. As Expert B remarked, “This technology shows promise as an assistive tool… which could help guide human investigators to focus on specific areas or elements they might otherwise overlook.” To fully validate the effectiveness of such collaboration, future research should include a comparative analysis of three distinct approaches: AI‐only analysis, human expert analysis, and combined AI–human analysis.

This complementary relationship could be particularly beneficial in resource‐constrained environments or time‐sensitive investigations. To evaluate potential cognitive bias risks, the experts first conducted independent analyses of all crime scene images before reviewing the AI‐generated results. This methodological approach provided an unbiased baseline for comparison and revealed essential insights about cognitive bias risks. The findings indicated that even with independent initial analysis, investigators might still be influenced by AI‐generated analyses in subsequent evaluations. Developing protocols to mitigate such biases will be crucial as law enforcement agencies move forward with AI integration in forensic work.

The analysis of AI error types reveals that misidentification of objects and missing critical evidence are the most common issues, accounting for 62% of all errors. This highlights specific areas where AI tools need improvement and where human expertise remains most crucial. The relatively low rate of errors in distance/size estimation (14%) suggests that AI tools perform well in spatial analysis tasks, indicating areas where AI could be particularly useful in supporting human investigators.

### 5.3 Ethical and legal considerations: Navigating new territory

The integration of AI in forensic science represents a paradigm shift that introduces specific ethical and legal challenges requiring practical consideration. The study's findings highlight three key areas of ethical concern that demand attention as these tools are implemented in forensic work:

First, the varying performance levels of AI tools across different crime scene types require careful consideration in forensic applications. While the study demonstrated stronger AI performance in analyzing homicide scenes (7.8) compared to arson scenes (7.1), this difference reflects the inherent complexity of fire‐related evidence analysis rather than AI bias. Arson scenes present unique analytical challenges, including evidence degradation from heat and water damage, complex chemical interactions, and multiple layers of scene modification. Understanding these technical limitations is crucial for appropriately deploying AI tools in different forensic contexts. This emphasizes the necessity of continuous validation and testing across diverse scene types and conditions, with clear guidelines for when AI analysis may be reliable.

Second, the “black box” nature of AI systems, as highlighted by Gunning et al. (2019), presents practical challenges in legal proceedings, where transparency is paramount. For AI‐assisted forensic analysis to be admissible in court, experts must be able to explain and justify their conclusions. This necessitates developing AI tools that not only assist in analysis but also provide clear, interpretable rationales for their suggestions ‐ a feature particularly important when these tools identify potential evidence or suggest investigative priorities. A critical consideration in implementing AI tools in forensic science is compliance with the Daubert standard for the admissibility of scientific evidence. The proposed approach of using AI as a decision support tool, rather than an autonomous system, may align with this standard's requirements. The system's outputs are testable, its error rates are quantifiable across different crime scene types, and the methodology maintains human expertise as the primary source for court testimony. This framework may ensure that AI‐assisted analysis meets legal admissibility standards while enhancing the investigative process.

Third, privacy and data security concerns emerge as critical considerations, particularly regarding the handling of crime scene images. The study's methodology highlighted the importance of proper data handling protocols, as even training images required careful management to protect privacy and maintain the chain of custody. As AI tools become more widespread in forensic work, robust frameworks for data protection, access control, and ethical image handling must be established.

The democratization of advanced forensic capabilities through AI tools presents both opportunities and risks. While these tools can enhance the capabilities of resource‐constrained agencies, they must be implemented with appropriate safeguards. This includes establishing clear protocols for tool validation, implementing user training requirements, and developing guidelines for appropriate use in different forensic contexts. The focus should remain on supporting, rather than replacing, expert judgment while maintaining the integrity of forensic investigations.

### 5.4 Practical implementation: Integrating AI tools into forensic workflows

The findings of this study suggest effective pathways for integrating AI tools within existing forensic workflows while maintaining the fundamental primacy of human expert judgment. Based on comprehensive expert feedback, a structured implementation framework emerged, highlighting the potential of AI as a complementary analytical tool across multiple stages of forensic investigation.

In the initial assessment phase, AI tools can analyze crime scene photographs and videos immediately after documentation to provide rapid preliminary evaluation. This post‐documentation analysis enables investigators to optimize their subsequent detailed examination strategy. This capability proved particularly valuable in complex scenarios with multiple evidence types, as demonstrated in homicide scenes where AI tools achieved high accuracy scores (mean 7.8). The AI analysis of scene photographs helps prioritize areas requiring focused investigation, serving as a valuable reference point before investigators begin their detailed examination.

The implementation framework extends to the detailed examination phase, where AI analysis functions as a supplementary verification mechanism, enhancing the thoroughness of scene investigation while preserving the critical role of expert judgment. This dual‐verification methodology synthesizes AI's systematic analytical capabilities with human expertise in evidence interpretation and context evaluation. The integration of AI support tools in documentation processes further streamlines reporting procedures while maintaining rigorous expert verification protocols.

Effective implementation necessitates established documentation protocols, specialized training programs emphasizing AI's supportive role, systematic performance validation, and integration with existing forensic platforms. This structured approach ensures that AI enhancement of forensic workflows occurs within a framework that prioritizes investigative integrity and maintains the essential primacy of expert analysis in forensic science.

### 5.5 Future directions and recommendations

The integration of AI in forensic science presents significant potential but requires careful attention to technological, legal, and ethical challenges to ensure its benefits are realized responsibly. Several key areas warrant focused attention:

1. *Specialized training for AI systems:* Developing AI models tailored to diverse forensic datasets can enhance performance across various types of crime scene investigations, including complex areas like arson analysis. Custom training will also help address inconsistencies in AI capabilities across different scenarios.
2. *Explainable AI (XAI):* The adoption of explainable AI systems, such as ChatGPT‐O1, which provide clear justifications for their outputs, is critical for improving transparency and aligning AI‐generated analyses with forensic and legal requirements. These systems enable investigators to better understand the reasoning behind AI recommendations, thereby increasing their trust and reliability.
3. *Standardization and best practices:* Establishing industry‐wide standards for the use of AI in forensic science is essential. These standards should encompass criteria for selecting appropriate tools, protocols for implementation, and guidelines for integrating AI‐generated insights with human expertise to promote consistency and reliability across jurisdictions.
4. *Cognitive bias mitigation:* Developing protocols to address potential cognitive biases stemming from interactions between human investigators and AI systems is crucial. Training forensic professionals to critically assess AI outputs will ensure balanced decision‐making and maintain the integrity of forensic investigations.
5. *Ethical framework development:* A robust ethical framework is necessary to address concerns related to privacy, data protection, and individual rights throughout the investigative process. This includes protecting the rights of all people involved ‐ witnesses, victims, suspects, and the general public whose data might be captured in crime scene documentation. This framework should guide the responsible use of AI in forensic science, balancing technological efficiency with the principles of justice and fairness.

The successful integration of AI in forensic science hinges on a cautious and measured approach underpinned by rigorous research, clear standards, and thoughtful implementation. Collaboration among researchers, practitioners, and policymakers is vital to fostering a system in which AI and human expertise complement one another to enhance investigative quality while adhering to ethical and legal standards. By addressing these priorities, AI can fulfill its potential as a transformative tool in forensic science.

## 6 CONCLUSION

The integration of AI into forensic science marks a significant step forward in criminal investigation techniques. Our study on the effectiveness of AI tools in forensic image analysis has revealed both promising potential and notable limitations of this emerging technology. With an average overall reliability rating of 7.4 out of 10, these AI tools demonstrate their capability to augment traditional forensic practices while highlighting areas for improvement.

The findings reveal a nuanced landscape of AI performance across various crime scene types, with performance variations between homicide scenes (7.8) and arson scenes (7.1). While this difference in performance scores is modest, it reflects the varying complexity levels in different forensic contexts. The data suggest that specialized AI training might help address the unique challenges of diverse crime scenes. The demonstrated capabilities in rapid observation and logical inference indicate the potential for accelerating initial crime scene assessments, potentially uncovering details that might elude immediate human detection.

However, the relatively lower performance of AI in evidence identification highlights that it is not yet capable of replacing human expertise in forensic science. Instead, the future likely lies in fostering a symbiotic relationship between AI and human investigators. Such collaboration could take several practical forms, including leveraging AI for rapid initial assessments of crime scenes to direct human investigators' attention to critical areas. AI tools could also be integrated into forensic training programs to enhance investigators' pattern recognition skills and improve overall efficiency. Furthermore, the development of AI‐assisted databases could facilitate cross‐referencing crime scene elements across cases, enriching investigative processes. To maximize the potential of this partnership, standardized protocols for AI–human collaborative analysis must be established, ensuring consistency and reliability in forensic procedures.

At the same time, it is essential to acknowledge the study's limitations and to outline directions for future research. While carefully selected and validated images from online sources enhance the ecological validity of our work, this approach may not fully capture the complexities of real‐world scenarios. The rapid evolution of AI technology further underscores the need for regular reassessment of tool capabilities. Future investigations would benefit from expanded datasets, including more diverse scene types—such as arson investigations that currently challenge AI tools—and the integration of AI analysis with other forensic technologies like mobile 3D scanning and chemical detection systems. Controlled comparative studies between AI‐assisted and traditional forensic teams are also warranted to quantify the impact on investigation quality and efficiency. Finally, establishing standardized validation protocols and conducting longitudinal studies tracking AI performance will be pivotal in refining these tools and ensuring their effective implementation in forensic work.

As we navigate this technological frontier, several key areas demand attention. Future development should focus on specialized AI models trained on diverse forensic datasets while simultaneously advancing explainable AI (XAI) technologies to enhance the transparency and interpretability of analytical processes. Equally important is the establishment of industry‐wide standards and best practices for AI integration into forensic workflows. This development must occur alongside the creation of robust ethical frameworks that comprehensively address privacy concerns, potential biases, and legal implications. These interconnected priorities will collectively shape the responsible evolution of AI applications in forensic science.

It is crucial to view these findings as preliminary steps in charting the future integration of AI in forensic work. As law enforcement agencies and forensic scientists continue to explore and refine these technologies, ongoing collaboration between technologists, forensic experts, legal professionals, and policymakers will be essential. By carefully navigating the challenges and opportunities presented by AI, we can work toward a future where technology enhances, rather than replaces, human judgment in the pursuit of justice.

In conclusion, while AI tools show immense promise in forensic image analysis, they must be deployed with caution and rigorous evaluation. The thoughtful incorporation of these technologies—alongside human expertise—can enhance accuracy, efficiency, and reliability in criminal investigations, ensuring that the pursuit of technological advancement fortifies, rather than compromises, the integrity of our justice system.

## CONFLICT OF INTEREST STATEMENT

The author declares no conflict of interest.
